# Video Validation of Tri-Axial Accelerometer for Monitoring Zoo-Housed *Tamandua tetradactyla* Activity Patterns in Response to Changes in Husbandry Conditions

**DOI:** 10.3390/ani12192516

**Published:** 2022-09-21

**Authors:** Sofía Pavese, Carlos Centeno, Lorenzo Von Fersen, Gabina V. Eguizábal, Luis Donet, Camila J. Asencio, Daniel P. Villarreal, Juan Manuel Busso

**Affiliations:** 1 Instituto de Investigaciones Biológicas y Tecnológicas (IIBYT), Consejo Nacional de Investigaciones Científicas y Técnicas (CONICET)-Facultad de Ciencias Exactas, Físicas y Naturales (FCEFyN)-Universidad Nacional de Córdoba (UNC), Av. Vélez Sarsfield 1611, Córdoba X5016GCA, Argentina; 2 Grupo de Investigación y Transferencia en Electrónica Avanzada (GInTEA)-Facultad Regional Córdoba (FRC)-Universidad Tecnológica Nacional (UTN), Maestro López esq. Cruz Roja, Córdoba X5016, Argentina; 3 Nuremberg Zoo, D-90480 Nuremberg, Germany; 4 Instituto de Ciencia y Tecnología de los Alimentos, FCEFyN-UNC Av. Vélez Sarsfield 1611, Córdoba X5016GCA, Argentina; 5 Parque de la Biodiversidad (ex Jardín Zoológico Córdoba), Rondeau 798, Córdoba X5000AVP, Argentina

**Keywords:** acceleration logging, activity, animal behaviour, ethogram, remote observation, telemetry, Pilosa, Xenartrha

## Abstract

**Simple Summary:**

In recent years, the use of technologies to remotely monitor animal behaviour has increased. However, when they are used for the first time in a new species, validation is needed. Therefore, a tri-axial accelerometer was used to monitor lesser anteater (*Tamandua tetradactyla*) behaviour. First, the influence of using a vest on the animals’ behaviour was evaluated. Second, correlations between video recordings and accelerometer data were made, allowing the researchers to obtain summary measures for each behaviour and a threshold to discriminate activity/inactivity events. Sensitivity and precision variables revealed the robustness of accelerometer monitoring. Third, animals responded to a reduction in enclosure complexity by increasing their activity. The relevance of the accelerometer was determined comparing data (activity level and activity cycle) with video recordings. The results indicate that an accelerometer attached to a vest is a reliable technology for monitoring the activity pattern of *T. tetradactyla* under semi-controlled environmental conditions.

**Abstract:**

Accelerometers are a technology that is increasingly used in the evaluation of animal behaviour. A tri-axial accelerometer attached to a vest was used on *Tamandua tetradactyla* individuals (n = 10) at Biodiversity Park. First, the influence of using a vest on the animals’ behaviour was evaluated (ABA-type: A1 and A2, without a vest; B, with a vest; each stage lasted 24 h), and no changes were detected. Second, their behaviour was monitored using videos and the accelerometer simultaneously (experimental room, 20 min). The observed behaviours were correlated with the accelerometer data, and summary measures (X, Y and Z axes) were obtained. Additionally, the overall dynamic body acceleration was calculated, determining a threshold to discriminate activity/inactivity events (variance = 0.0055). Then, based on a 24 h complementary test (video sampling every 5 min), the sensitivity (85.91%) and precision (100%) of the accelerometer were assessed. Animals were exposed to an ABA-type experimental design: A1 and A2: complex enclosure; B: decreased complexity (each stage lasted 24 h). An increase in total activity (%) was revealed using the accelerometer (26.15 ± 1.50, 29.29 ± 2.25, and 35.36 ± 3.15, respectively). Similar activity levels were detected using video analysis. The results demonstrate that the use of the accelerometer is reliable to determine the activity. Considering that the zoo-housed lesser anteaters exhibit a cathemeral activity pattern, this study contributes to easily monitoring their activities and responses to different management procedures supporting welfare programs, as well as ex situ conservation.

## 1. Introduction

In recent years, the application of technologies to remotely monitor animal activity (e.g., accelerometers) have made it possible to increase sampling capacity, with minor sampling effort and observer bias [1,2,3,4,5]. Tri-axial accelerometers are sensors that simultaneously measure the acceleration of an object/organism in space along three dimensions, X, Y and Z [6]. These devices offer information that can be processed to identify animals’ activity states [7]. In brief, information is reflected by two main components: static acceleration, related to the force of gravity and the orientation of the accelerometer in space, and dynamic acceleration, associated with the movement of an animal [8]. Normally, accelerometers are mounted to the animal’s body with a device that is designed for such use (e.g., a collar or vest), but this does not affect its behaviour. The effect of mounting the accelerometer varies considerably according to the taxon and the technique used [9], and it has been studied in several groups [10,11,12].

When researchers use an accelerometer to determine activity and behavioural patterns in a species for the first time, validation is a key concern [13,14]. If accelerometers are used without previous validation, the robustness of the results and replicability of experiments are compromised. Brown et al. [13] indicate that the validation process can be carried out by synchronising behavioural observations (i.e., video recordings) with simultaneously obtained data from the accelerometer. From this point, it is possible to classify accelerometer data variability more precisely, using manual methods, statistical algorithms or even specific software [13].

*Tamandua tetradactyla* (Mammalia: Pilosa: Myrmecophagidae) is a medium-sized anteater species endemic to South America. The Myrmecophagidae family includes *Myrmechophaga trydactyla* (giant anteater) and the Tamandua species: *T. tetradactyla* (lesser or southern anteater) and *T. mexicana* (northern anteater) [15,16,17]. The lesser anteater conservation status varies according to geographical scale. The animals are threatened in Córdoba (Argentina) [18], near threatened in Argentina [19] and of little concern globally [20].

Many aspects of *Tamandua* spp. biology remain to be elucidated [21,22], and their activity and behavioural pattern are no exceptions [17,23]. In the wild, *T. tetradactyla* individuals have been observed to be active during both light and dark phases. They show mainly arboreal habits, although they also rest, feed and move in the ground [17,24,25]. In captivity, similar observations were made in previous studies using infrared cameras [26,27]. Considering that behavioural patterns are adaptive responses determined by individual intrinsic characteristics (e.g., physiology and behaviour), as well as external factors, validating the accelerometer will allow to us to expand our knowledge on *T. tetradactyla* behaviour. The present study was developed in light of the general hypothesis that the use of a tri-axial accelerometer would enable the characterisation of the activity pattern of zoo-housed *T. tetradactyla* without affecting it. Therefore, the purposes of this study were to: (1) evaluate the effect of a vest-mounted accelerometer on the general activity pattern; (2) correlate accelerometer and video recording information, evaluating fluctuations of the X, Y and Z data series (accelerometer) in relation to changes in the orientation of the animals’ movements (video); and (3) characterise the activity pattern during husbandry changes using an accelerometer.

## 2. Materials and Methods

### 2.1. Studied Animals and Housing Conditions

Ten adult *Tamandua tetradactyla* (henceforth referred to as ‘lesser anteaters’; n = 5 females and 5 males; average weight: 6.5 ± 0.8 and 8.5 ± 0.6 kg, respectively) were studied at Biodiversity Park (former Córdoba Zoo, 31°12.32′ S; 64°16.84′ W), Córdoba, Argentina. Lesser anteaters were housed in individual contiguous enclosures, maintained under natural conditions of photoperiod, temperature and humidity. A diet was administered following the Manual of Nutrition and Diet for Wild Animals in Captivity [28]. Additionally, considering that food-based environmental enrichment has proven useful in favouring the expression of natural behaviours in this species [29], other food items were offered (e.g., honey, ants, and fruits).

**Figure 1:**
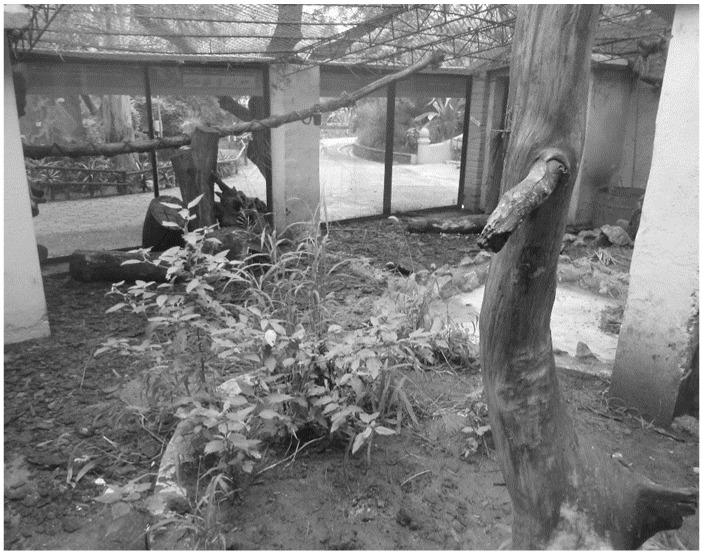
Housing of adult lesser anteaters (*Tamandua tetradactyla*) at Biodiversity Park. Enclosures were under natural conditions of photoperiod, temperature and humidity. Image shows the view from the rear of enclosure 2.

Housing conditions were characterized by enclosures designed following recommendations for the species [21,30]. All enclosures were similar in physical characteristics and size, and were located next to each other in the same area of the institution; more details can be found in the work of Eguizábal et al. [26]. In brief, three walls were made of concrete, and the front wall was made of glass (allowing public view), while the roof was made of wire mesh (10 × 10 cm) and the floor of soil and wooden substrate (Figure 1). To achieve the possible beneficial effects of providing new exploration opportunities, animals were rotated between enclosures every 2 weeks.

### 2.2. Video Cameras and Tri-Axial Accelerometer

Behaviour was continuously monitored using infrared video cameras (HIKVISION Turbo HD- IR Turret Camera- DS 2CE56C2T IRM) and recorded using a digital video recorder (HIKVISION Turbo HD DVR- DS 7200 Series). All observations were made by the same researcher (S.P.).

**Figure 2:**
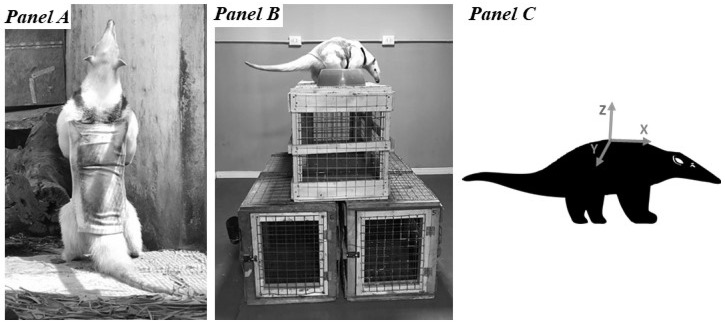
Photographs and illustration of adult lesser anteaters (*Tamandua tetradactyla*) at Biodiversity Park during Studies 1 and 2. Panel A: Male in the enclosure with the accelerometer-mounting accessory (vest). Panel B: Male on the climbing structure in the experimental room (Study 2). The two feeders, one in the lower left part and the other in the upper part, are shown. Arrows indicate the two feeders. Panel C: Diagram that represents the position of the X, Y and Z axes of the accelerometer in relation to the body of a lesser anteater. The vectorized image was specially designed to this study.

The accelerometer was a Techno SmArt tri-axial-AXY-3 device [31]. It weighed 0.7 g, and its dimensions were 9 × 15 × 4 mm. The equipment was configured for continuous sampling at a frequency of 10 Hz (10 data/second/axis) and with a dynamic range of 8 g. The accelerometer was placed in the pocket of the vest, positioning the X axis in correspondence with the anterior–posterior body axis, the Y axis with the right/left and the Z axis with up/down (Figure 2, Panel A).

The software to carry out accelerometer monitoring was specially designed during this research. The software was developed with LabVIEW (National Instruments) in the Laboratory of the Advanced Electronics Research and Transfer Group (Córdoba Regional Faculty of the National Technological University). The behavioural activities observed in the videos and the series of data obtained from the accelerometer were analysed simultaneously.

With this software, for each study, it was possible to simultaneously visualize the stored acceleration data (Y axis) in the time domain (X axis) in an X–Y graph, and the video recorded of the animal with the vest and accelerometer in a video viewer included on the same screen. The start time of each of the studies was used to synchronize the acceleration data and the corresponding video in the application software. Then, using cursors available in the software, it was possible to characterise the different behavioural activities, by going through the X–Y acceleration graph and simultaneously modifying the time instant in the associated video. Additionally, the software included controls that allowed us to work with the acceleration signals, applying zoom, or including or removing any of them from the X–Y graph.

### 2.3. Study 1—Effects of a Vest on Activity Pattern

The vest was specially designed for this study, considering lesser anteater measures. The chosen textile was Scuba, a low-density neoprene-like material, which was printed using a heat-transfer technique to imitate the fur of the species. The vest had a pocket to mount the accelerometer on the dorsal area of an animal’s back (i.e., between the shoulder and the pelvic girdle). The vest weighed 64.0 g, which corresponded to 0.7 and 1% of the average body weights of males and females, respectively (Figure 2, Panel A). In this study, the accelerometer was not placed in the vest pockets, since it was considered unnecessary to expose the machine in the test and due to its low weight (0.7 g: 0.009% of the animals’ average body weight).

To evaluate the effect of the vest on which the accelerometer was mounted, the activity pattern was assessed through video analysis. The activity pattern of lesser anteaters was studied using an ABA-type experimental design over 3 consecutive days in May 2019. This experimental design was chosen due to the low number of lesser anteaters available (N = 6) at that moment in the institution. In this design, each individual acted as its own control when subjected to an experimental treatment [32]. Sampling began on day one in order to obtain behavioural baseline data without the vest (stage A1); on day two, with the vest on (stage B); and ended the day after, without the vest (stage A2). It should be noted that the study started at 14:00 h, when lesser anteaters received their daily food ration and were usually active. Thus, each experimental day started at 14:00 h and ended at 13:59 h of the following calendar day.

The activity pattern of each lesser anteater was continuously monitored using a video camera located in the enclosure. The animals’ activity was sampled using the focal animal sampling method [33], registering *activity events* (records/day), *total activity* (hours/day) and *activity per hour* (percentage/hour). Each *activity event* started when an active behaviour (e.g., feeding, exploration, and locomotion) was registered, and ended when rest behaviour (the only inactive behaviour) was detected [26]. The resolution time was 1 s, and any event that lasted less than 1 s was considered irrelevant.

For the statistical analysis, *activity events* were analysed using a mixed general linear model (MGLM), and a Poisson error distribution was assumed. ABA stages (A1, B, and A2) were included as a fixed term, and sex and individual were random factors. The same factorial structure was used for *total activity* statistical analysis, but with a mixed linear model (MLM). *Activity per hour* was not subjected to statistical analysis due to its descriptive nature. When data did not meet a normal distribution, a natural logarithm transformation was applied.

### 2.4. Study 2—Video Analysis Validation of the Accelerometer

In order to evaluate fluctuations in the X, Y and Z data series (accelerometer) in relation to changes in the orientation of the animals’ movements (video), each lesser anteater (N = 10) was subjected to a 20 min test in an experimental room during August–September 2019. The experimental room (surface: 3 × 3 m; height: 2.4) was located in the laboratory next to the animal enclosures; more details can be found in the work of Zárate et al. [34,35]. The room had 4 homogeneous walls, a cement floor and a single entrance through a door, and was under controlled lighting and temperature conditions. In order to stimulate the expression of different behaviours, the experimental room had a climbing structure made of wood and wire mesh (vertical movement and climbing), and one feeder located on the floor and one feeder located above the climbing structure (feeding) (Figure 2, Panel B). These elements in the experimental room were provided to motivate a high number of different behaviours. It was expected that the behaviours registered in the videos were associated with different values of acceleration.

Prior to starting the test, the vest was mounted on each lesser anteater housed in the enclosure, and then, in the experimental room, the tri-axial accelerometer was placed in the pocket of the vest (Figure 2, Panel C). The test began after a 5 min acclimation period per animal, and a food ration was administered by two feeders after 10 min. Animal behaviour was simultaneously monitored by two means: accelerometer and video recordings. Both devices were temporally synchronized using their software. Video recordings were obtained via two cameras located in the ceiling of the experimental room, one in a central position and one in a corner.

The animals’ behaviour was sampled using a focal sampling method and employing a continuous recording rule [33]. First, through video analysis, we registered *behavioural events* using a previously developed ethogram [26]; inactive behaviour: resting; and active behaviours: motionless, feeding, locomotion, descent locomotion (or climbing down), self-grooming, ascendent locomotion (or climbing up), inverted locomotion (inverted climbing), exploration, alert, repetitive locomotion, and others. Then, we classified the same *behavioural events* in the serial data of the accelerometer (X, Y, and Z values), considering the beginning and the end of each *behavioural event* registered in the video. The X, Y and Z variables (acceleration values for each respective axis) and the overall dynamic body acceleration (ODBA) were considered according to Ladds et al. [36]. In order to obtain the value of the dynamic acceleration (m/s^2^) of each axis, static acceleration was calculated as a 3 s average and then subtracted from each of the axes [37].

Table S1 in the Supplementary Material, reports the summary measures obtained for the variables X, Y, Z and ODBA for each behaviour. These measures were used in the exploratory analysis to characterise the resulting pattern of fluctuations associated with the behaviours. Following the simplest approach for acceleration waveform statistics utilized frequently by others researchers [13], such as Mean, Minimum, Variance, among others, we were not able to establish criteria to discriminate active behaviours (e.g., locomotion vs. repetitive locomotion). Otherwise, ODBA variance was more useful than X, Y, Z variances to discriminate *behavioural events* classified as active and inactive. In order to explore this parameter (ODBA variance), a cluster analysis using the average Euclidean distance (non-hierarchical K-means method) was used [13]. The threshold was established at 0.0055, since it was observed that the active behaviours had greater or equal values.

To test ODBA variance in real context, a 24 h pilot test was performed during October 2019. The accelerometer was mounted on one lesser anteater in its enclosure, and the animal’s behaviour was monitored for 24 h using two cameras (one positioned in the enclosure and one in the shelter). Active and inactive behavioural events were recorded continuously; as an example, one active event may include the expression of different behaviours. Each *activity event* started when an active behaviour (e.g., feeding, exploration, or locomotion) was registered, and ended when rest behaviour (the only inactive behaviour) was detected [26]. Each event observed in the video recordings was associated with the corresponding accelerometer data series. Using these data, a matrix was obtained comparing the number of observed events in the video with those predicted using the accelerometer (according to the threshold value 0.0055). The precision and sensitivity percentages were calculated based on this matrix: precision = true positive / (true positive + false positive); sensitivity = true positive / (true positive + false negative). True positive: coincidence in observed event using video recordings and classification of event using the accelerometer; false positive: observed inactive event using video recordings and classified using the accelerometer as an active event; false negative: observed active event using video recordings and classified using the accelerometer as an inactive event [38,39].

### 2.5. Study 3—Assessment of Biological Relevance of Using Accelerometer to Monitor Activity Pattern

**Figure 3:**
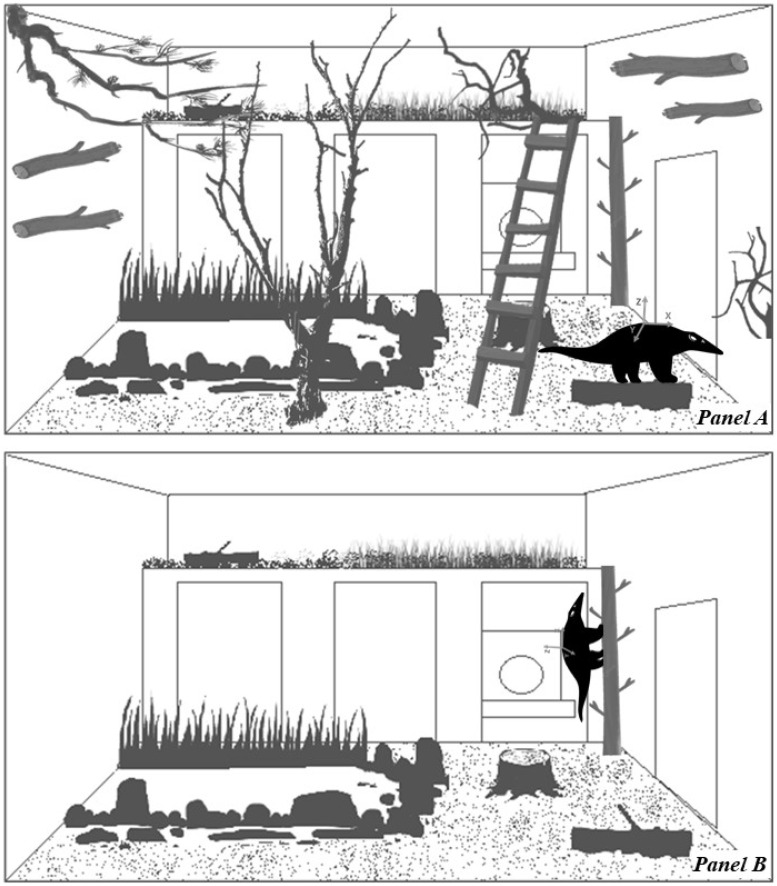
Diagram representing the enclosures used during Study 3 to evaluate behavioural response of adult lesser anteaters (*Tamandua tetradactyla*) to enclosure complexity. An ABA-type experimental design was carried out. Panel A: complex enclosure with climbing structures (stages A1 and A2). Panel B: enclosure with reduced complexity (stage B).

The activity pattern and activity level (the proportion of time that animals spend active) are behavioural information that can provide an indicator of how animals respond to changes in their environment. To evaluate the biological relevance of using an accelerometer to measure the activity level of lesser anteaters (N = 6), animals were studied in an ABA-like experimental design [32] for three consecutive days in October 2019. Lesser anteaters were studied on: day one in a routine housing condition (stage A1: baseline), day two in an enclosure with reduced complexity (stage B: treatment), and day three with their housing condition restored (stage A2). During stages A1 and A2, the complex enclosure contained a wooden shelter, several climbing structures (e.g., logs, stairs, and wire mesh ceiling), plants, and soil and wood substrate (Figure 3, Panel A). During stage B, lesser anteaters were rotated (<15 s of handling transportation) to the contiguous enclosure, in which most climbing structures were eliminated (Figure 3, Panel B).

Activity pattern was simultaneously monitored by two means: an accelerometer and video recordings. The active and inactive behaviours (records/day) of each lesser anteater were continuously monitored using two video cameras located at the enclosure, via the ethogram employed in Studies 1 and 2. First, the animals’ activity was recorded on a video camera at 5 min intervals using an instantaneous sampling method [40]. Based on previous studies [33,40,41], activity was measured for 9 s at each sampling point (1 record/sample point). A total of 288 frequency records were obtained per individual each day. Second, the activity was then associated with the obtained accelerometer data, calculating the ODBA for each sampling point (5 min intervals). After this, ODBA variance was obtained, which allowed us to classify the values of 288 sampling points per stage as active or inactive events depending on whether they exceeded the ODBA threshold.

In addition, the software ActogramJ [42] was used to build actograms based on active/inactive records from both the videos and accelerometer data. Actograms represent the way that the animals’ activity is distributed throughout the day, allowing us to ascertain their activity patterns. Using the individual actograms, smoothed mean actograms were then constructed for each of the ABA stages. It was also possible to calculate the *acrophase* (time of the day of peak activity) and *start/end of activity* (time) using both technologies.

For statistical analysis, based particularly on Study 2, it should be clarified here that the objective of the study was to measure activity/inactivity, and not detailed behaviours. First, the effect of treatment on activity patterns was analysed, considering the information obtained using the video recordings and accelerometer analysed. A mixed general linear model (MGLM) was used, and a Poisson error distribution was assumed. ABA stages (A1, B, and A2) were included as a fixed term, and sex and individual characteristics were random factors. Then, a T-test was employed to compare the results of the activity level per stage obtained using the camera and accelerometer. Additionally, a T-test was also employed to compare the values of the *acrophase*, *start* and *end of activity* obtained using both previous methodologies.

Again, a matrix for percentages of precision and sensitivity was obtained by comparing the number of active events observed in the video recordings with those predicted using the accelerometer.

### 2.6. Statistical Analysis

For all studies, normality was verified using the modified Shapiro–Wilks test, and variance homogeneity was verified using the F-test of equality of variances. For a posteriori tests, Fisher’s test was applied when the statistical analysis showed a *p*-value ≥ 0.05. All analyses were performed using InfoStat [43]. The significance level was 5% for all tests. Values are reported here as the mean ± SEM unless otherwise noted.

## 3. Results

### 3.1. Study 1—Effects of Accelerometer Mounting Using a Vest

**Figure 4:**
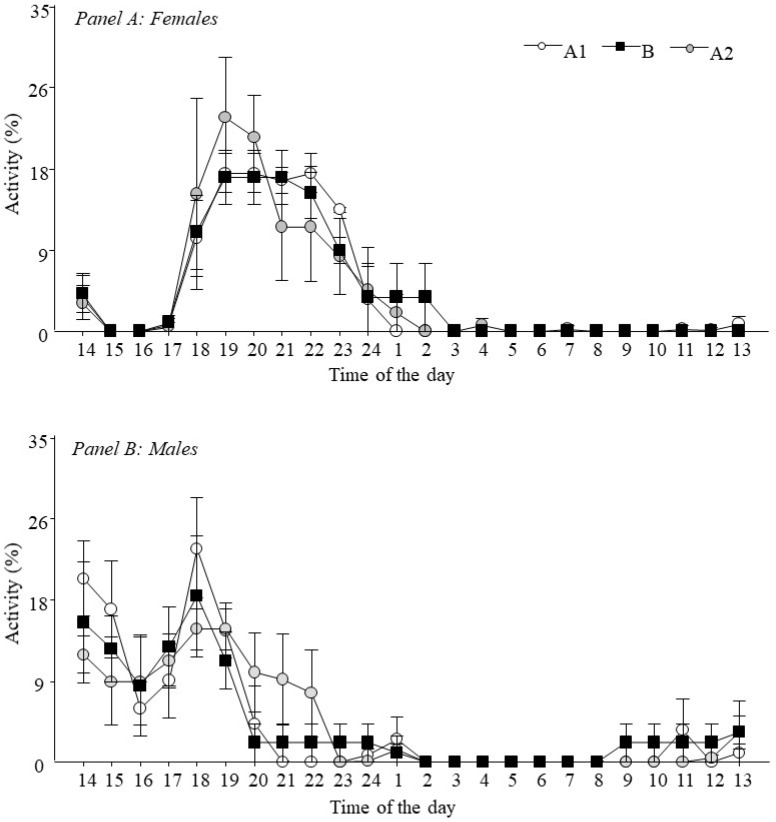
Percentage of activity per hour of adult *Tamandua tetradactyla* (n = 3 females, Panel A; 3 males, Panel B) exposed to semi-controlled conditions during ABA stages. Behaviour was recorded continuously for 3 consecutive days (A1: baseline, without vest; B: treatment, vest on; A2: posterior, without vest). The beginning of the X axis corresponds to feeding time.

No changes during ABA were detected for *activity events* (A1 = 3.67 ± 0.88; B = 2.50 ± 0.22; A2 = 2.50 ± 0.43 records/day) or *total activity* (A1 = 5.68 ± 0.79; B = 7.34 ± 1.93; A2 = 6.15 ± 0.98 h/day). Figure 4 shows the activity per hour (percentage/hour) according to ABA stages for both sexes.

### 3.2. Study 2—Correlations between Data from Accelerometer and Video Recordings 

**Figure 5:**
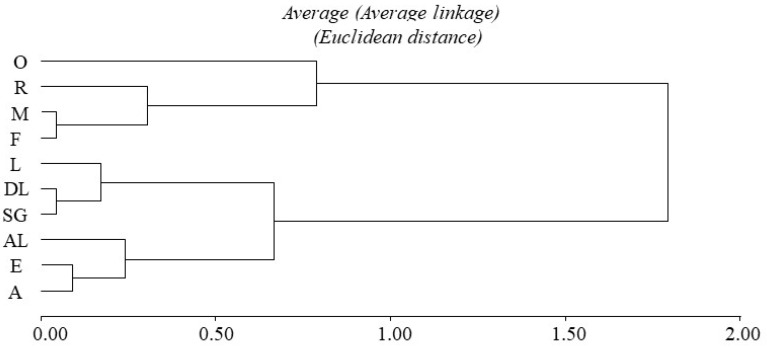
Cluster analysis for the ODBA variance values associated with the behaviours of adult *Tamandua tetradactyla* (n = 5 females and 5 males) exposed to test in the experimental room. The hierarchical sequence of cluster formation was produced taking into account the average comparison. Abbreviations: O: others; R: resting; M: motionless; F: feeding; L: locomotion; DL: descent locomotion; SG: self-grooming; AL: ascendent locomotion; E: exploration; and A: alert.

**Figure 6:**
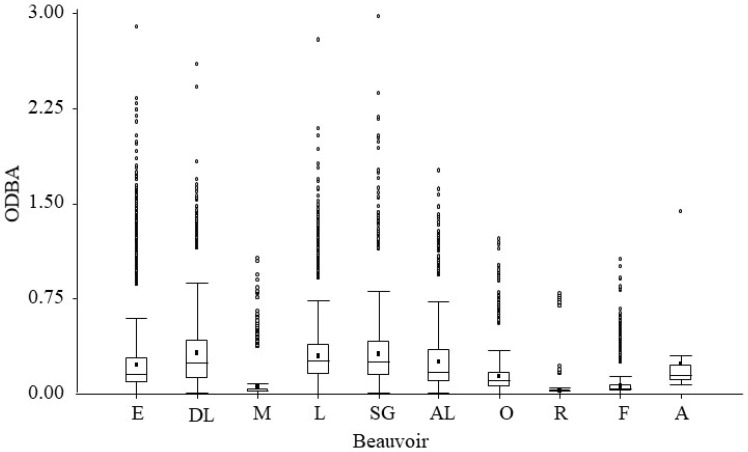
Overall dynamic body acceleration (ODBA) variance values of behaviours of adult *Tamandua tetradactyla* (n = 5 females and 5 males) exposed to test in the experimental room. Behaviour was monitored continuously by means of a tri-axial accelerometer. Box plots show median values (horizontal line), average (dark square dot), 25th and 75th percentiles (bottom and top lines), SD ± 1 (whiskers) and values outside the range (black circles). Abbreviations: O: others; R: resting; M: motionless; F: feeding; L: locomotion; DL: descent locomotion; SG: self-grooming; AL: ascendent locomotion; E: exploration; and A: alert.

The cluster analysis for ODBA variance values is shown in Figure 5. Behaviours with lower acceleration values (rest, feeding, motionless, and others) have a common origin in the same node, in contrast to the rest. Moreover, these behaviours have an ODBA variance value close to 0, while the others have higher values (Figure 6).

**Figure 7:**
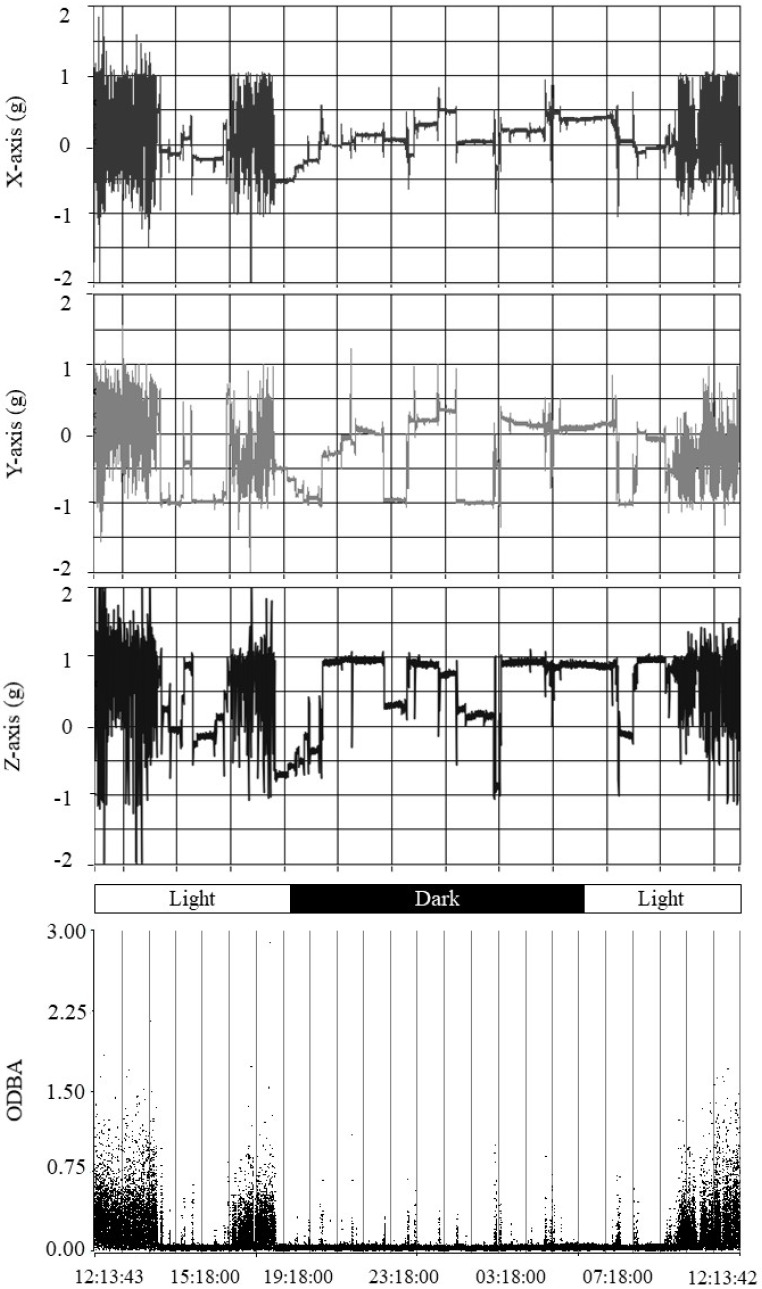
Profiles of the accelerometer data series (Panel A) and ODBA dot plot (Panel B) obtained during the 24 h pilot test carried out on an adult *Tamandua tetradactyla* individual exposed to semi-controlled conditions in its enclosure. Panels A–C: X, Y and Z axes. Panel D: vertical lines indicate 60 min periods throughout the test. Light and dark periods associated with light/dark photoperiodic phases are shown.

During the complementary pilot test, video analysis allowed the determination of 71 activity and inactivity events on one animal housed in the enclosure. Fluctuations in the positional axes and corresponding ODBA values detected over a 24 h period using the accelerometer are illustrated in Figure 7 (Panels A and B, respectively).

**Figure 8:**
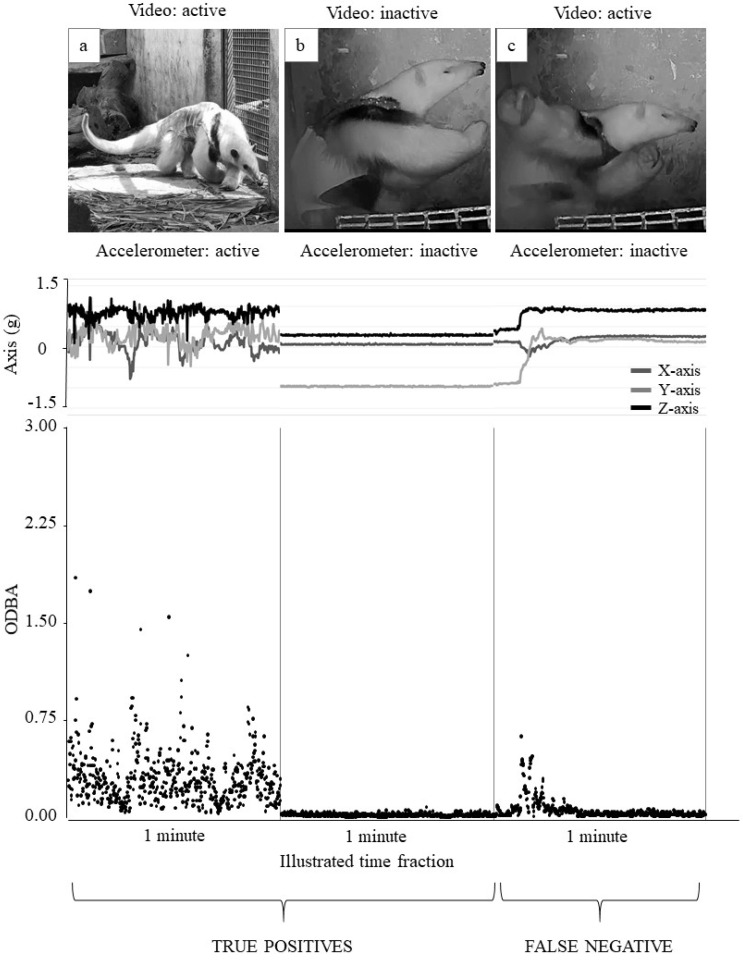
Comparison of a fraction of behavioural events observed using video recordings and predicted using an accelerometer. Data are from the 24 h pilot test carried out on an adult *Tamandua tetradactyla*. In the upper part, images show a frame of 1 min of video recordings. Images a and c show a lesser anteater that was active (locomotion and others), and image b shows an inactive state of the same animal (rest). In the middle part, X, Y, and Z profiles are shown for the same fractions of the video. In the lower part, detected ODBA variance values obtained from accelerometer monitoring for each behavioural event are illustrated. According to activity threshold value (0.0055), a was classified as active (true positive) and b and c as inactive (true positive and false negative, respectively).

During the complementary pilot test, addition information was obtained to support validation. By associating the data obtained from the video recordings and accelerometer, it was possible to compare the number of observed events in the videos with those predicted using the accelerometer: 26 active events (true positive), 35 inactive events (true positive), 10 active events classified as inactive (false negative) and 0 inactive events classified as active (false positive). These results correspond to a 100.00% precision and an 85.91% sensitivity rate. Examples of true positives and a false negative are shown in Figure 8 (time scale is not associated with the length of each event).

### 3.3. Study 3—Characterisation of Behavioural Response to Changes in Enclosure Complexity Using an Accelerometer

**Figure 9:**
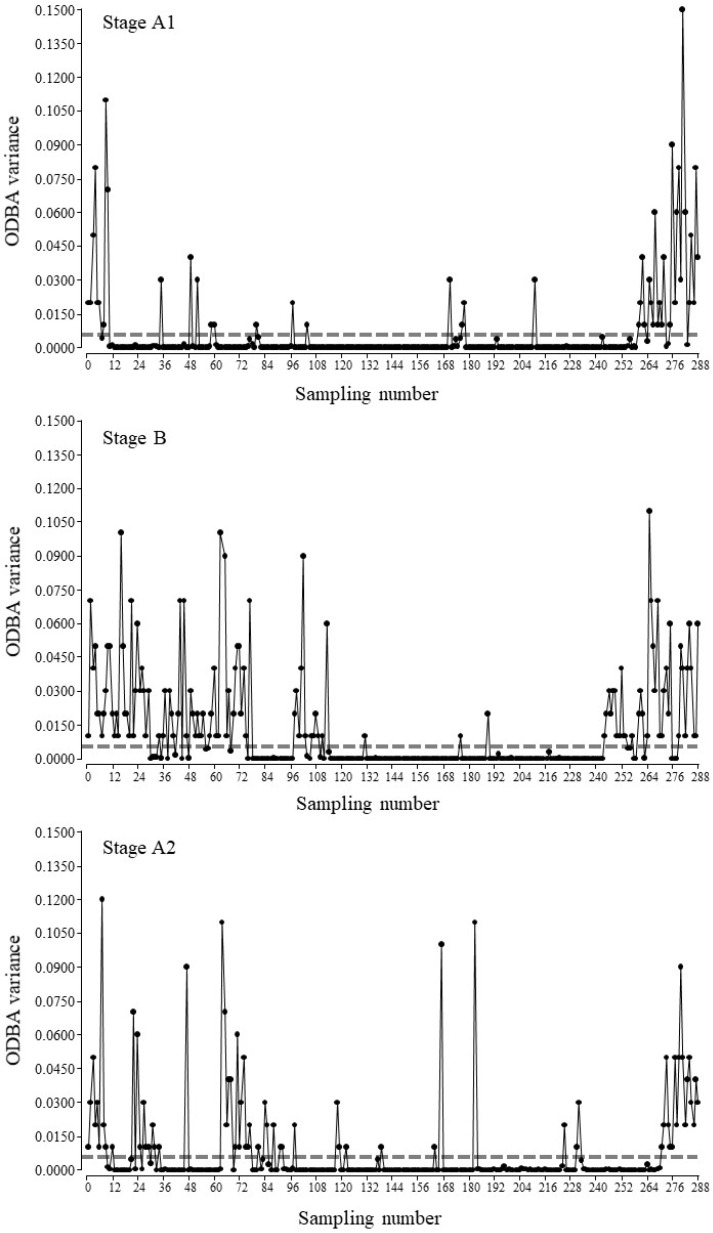
Activity pattern of individual *Tamandua tetradactyla* (as an example) exposed to semi-controlled conditions in response to enclosure complexity. Experimental stages: during A1 and A2, lesser anteaters were in the complex environment, and during B, they were in the enclosure with reduced complexity. ODBA variance was calculated for each sampling point (90 records/sampling; 12 samplings/hour). The horizontal dotted line indicates the threshold value to discriminate active behaviours from inactive ones.

When analysing the effect of enclosure complexity on total activity, variations were detected for video recording data (B > A2 > A1 records/day; F_2,46651_ = 161.4; *p* < 0.0001). The percentages of activity for these stages were 35.36 ± 3.15; 29.29 ± 2.25, and 26.15 ± 1.50, respectively. The same pattern was also observed in the study of ODBA variance data obtained from the accelerometer (B > A2 > A1 records/day; F_2,5181_ = 26.1; *p* < 0.0001; Figure 9; an example of an individual profile).

By linking the data from the video recordings and the accelerometer, it was possible to compare the number of events observed in the video recordings with those predicted using the accelerometer: 1121 active events (true positive), 3613 inactive events (true positive), 450 active events classified as inactive (false negative) and 0 inactive events classified as active (false positive). These results correspond to a 100.00% precision and a 91.30% sensitivity rate.

Finally, comparing *total activity* between the two technologies, significant differences were found in two of the three stages: the A1 and A2 stages (A1: T = −5.22, *p* = 0.0004; A2: T = −2.24, *p* = 0.0486). In both cases, the averages obtained using the accelerometer were higher than those of the video analyses. In addition to this, no statistical differences were detected between technologies when the acrophase (T = 0.84, *p* = 0.4334), start (T = 0.49, *p* = 0.6332) and end of activity (T = −0.15, *p* = 0.8833) were compared. For instance, the acrophase assessed using video recordings was 6.17 ± 1.14 h, and using the accelerometer, it was 8.91 ± 3.05 h.

## 4. Discussion

The use of a tri-axial accelerometer was validated to assess activity patterns in adult *Tamandua tetradactyla* under human care. In Study 1, the effect of mounting the accelerometer using a vest on the animals’ activity was discarded as a source of variation in the activity pattern. This result enabled its use to monitor activity in Studies 2 and 3. During Study 2, it was possible to correlate the fluctuations in axis activity obtained using the accelerometer with the behaviours observed in video recordings. Based on Study 2, after various statistics and their values in relation to the detected behavioural activities were explored, the ODBA variance was determined as a threshold parameter that allowed for discrimination between activity and inactivity events; further research is necessary to categorise different behaviours. Finally, during Study 3, findings revealed that a tri-axial accelerometer was useful to monitor the responses of lesser anteaters to changes in environmental complexity by increasing their activity in the less complex enclosure.

Given the great potential that can be attributed to accelerometers, a first step in applying them to a new species is to find a suitable mounting device that minimally affects the behaviour of the animals [44]. With this in mind, a vest was designed specifically for use on *T. tetradactyla* individuals to allow the proper and secure positioning of the accelerometer on the animals’ bodies. The vest allowed us to keep the X axis aligned with the longitudinal body axis, with no apparent rotations or changes in position. No obvious short-term changes were observed in the animals’ activity throughout the day when using the vest (i.e., activity cycle). In fact, in the authors’ observations, aversive or disturbed behaviours were detected. However, more studies are necessary to assess the long-term effects of using vests on animals, such as thermal discomfort and/or skin damage. In addition, one problem with the design of the vest was material durability, as it was observed that some individuals damaged the vest with their claws during self-grooming, so it might be interesting to explore new textile materials that fit their bodies just as well but are more durable. Nevertheless, in relation to the other mounting devices that have been used for studying tamanduas and giant anteaters’ behaviour (*Myrmecophaga tridactyla*), the vest could be a more appropriate mounting accessory, avoiding anaesthesia and the use of rigid elements that could harm individuals [45,46]. Thus, the vest could also be a tool for attaching other small devices, such as GPS tracking systems, magnetometers, further broadening the range of behavioural studies in zoos or animal facilities [4,13,47].

During Study 2, detailed information regarding the movements and positions of lesser anteaters’ bodies for most behaviours was obtained from accelerometer monitoring. Only the behaviours of *repetitive locomotion* and *inverted climbing* were not observed in the experimental room, and had to be further characterised in the 24 h pilot test. From cluster analysis, a first approach was made, and it was determined that behaviours with low dynamic acceleration (ODBA variance close to 0; *rest*, *motionless* and *feeding*) and minor variations in the X, Y and Z axes had a common node. Furthermore, the associations between acceleration profiles and behavioural activities observed in video recordings revealed that the behaviours that resulted in least variations in the values of the axes involved fewer body movements. The same results in terms of *rest* behaviour have been found in studies of other species using accelerometers [1,39]. The lesser anteaters studied ingested artificial food with minimal body movements, helping with their tongue and/or claws but without large displacements, for which there were no marked fluctuations in acceleration values. It is worth noting that artificial food (i.e., a semi-liquid balanced diet) is very different from what the species can find in nature (i.e., social insects). However, when offering other feeding resources, which are frequently administered in food-based environmental enrichment at zoos, the fluctuations in acceleration values could be greater. It would be interesting to explore the relationship between preys and *T. tetradactyla* individuals using an accelerometer, considering that the ODBA variance parameter is useful to assess energy expenditure [13,48].

After various statistics and their values in relation to the detected behavioural activities were explored, the ODBA variance was determined as a threshold parameter that allowed for discrimination between activity and inactivity events. This threshold proved to be robust, with high percentages of precision and sensitivity during both Studies 2 and 3. Further video exploration revealed that the false negatives were events in which an animal interrupted its resting behaviour by opening its eyes or adjusting its position to continue resting. Therefore, acceleration was very low, and behaviour was misclassified as rest according to the accelerometer records. When active behaviours were taken into account, a wide range of ODBA variance values were detected, especially for the different types of *locomotion*, so they could not be distinguished from each other using this parameter (or with the other statistical parameters analysed). Perhaps this is because the ODBA variance is affected by variables such as the mean, the sampling frequency, and the resolution of the accelerometer [37]. Considering that ODBA is an important parameter to assess energy expenditure, and that its combination with other measures can contribute to the integration of biomechanics, behaviour and ecology [13,48,49], further studies should focus on its application to distinguish active behaviours in *T. tetradactyla*. It is also important for further studies in *T. tetradactyla* to take into account that the ODBA parameter was useful to discriminate between active behaviours when analysis was performed using automatic machine learning [39,50,51]. This was demonstrated in other studies employing other parameters (such as static lateral acceleration) and processing accelerometer data through the automatic classification of active behaviours [52,53]

During Study 3, the use of the accelerometer proved its biological relevance in characterising the activity pattern of *T. tetradactyla* individuals in enclosures with different complexities of climbing elements. The analysis of both video and accelerometer data indicated that, in the environment with reduced complexity, lesser anteaters were more active. As it was not possible to define a threshold to discriminate between behaviours, future studies are necessary to detect if animals perceive husbandry changes as positive (e.g., increased exploration) or negative (e.g., increased repetitive locomotion). Furthermore, when animals returned to the complex enclosure (A2 stage), they were more active than in the basal stage (A1), perhaps indicating that the treatment effect lasted. There is limited information on this subject, but a case study showed that a greater expression of active behaviours was generated when two *T. tetradactyla* individuals were subjected to different types of enrichment [54]. Similarly, in the present study, the reduced environmental complexity could have acted as a novel environment, fostering the increased activity and perhaps the ability to deal with a new situation. This interpretation is supported by the fact that no increases in the expression of abnormal behaviours or stereotypies were observed (in the videos).

In the wild, it has been found that 47% of the mammals that inhabit the forests of Brazil (one of the current distribution sites of *T. tetradactyla*) have changed their period of activity in response to different human disturbances [55]. For example, *Dasypus novemcinctus* (a xenarthra-related species) has been found to increase its diurnal activity with habitat fragmentation [56]. The authors of previous studies estimate that this could be due to factors such as stress or difficulties in acquiring food, and hypothesise that the flexibility in activity patterns contributes to the ability of this species to adapt to changes induced by human activities. Although no evident changes in the activity cycle (the acrophase, start and end of activity) were found for *T. tetradactyla* individuals, the increased activity in the least complex environment could be understood as a positive response to environmental changes. In the context of zoological institutions, we consider a beneficial reaction of individuals, since frequently the lack of behavioural response is linked to a chronic state of stress.

When comparing *total activity* values obtained using both methodologies, more records per day were observed in the videos (during A1 and A2). This difference could be explained by the sensitivity of the accelerometer monitoring, which, despite being high (91.3%), indicated that not all active events were detected as such. For example, on several occasions when animals were *feeding*, they were almost immobile, and only extended and retracted their tongues, causing this behaviour to be misclassified as *rest*. Moreover, as mentioned above, some false negative events arose when animals briefly interrupted their resting behaviour (opening their eyes, changing their position, etc.), which indicates an underestimation of their activity, albeit a biologically irrelevant one. Other studies have shown that accelerometers are useful for clearly discriminating between resting behaviour and active behaviours. However, the reduced performance of accelerometers is evident based on accuracy and precision parameters when they were applied to monitor behaviours with low acceleration, such as feeding and other movements [53,57,58]. Nevertheless, when researchers want to analyse results, it is also important to consider the position of the accelerometer on the animal body (e.g., ear or neck) and/or the species-specific feeding strategy; for example, results on feeding behaviour collected using accelerometers mounted on the necks of cattle will be different for grazing animals in a field vs. animals in a feedlot [38,59].

There are some practical aspects to consider when using the accelerometer to monitor the behaviour of the lesser anteater: (a) donning the vest requires three or four trained individuals and can be completed in a short time (<5 min); (b) in contrast, the accelerometer can be easily attached and removed by a single person; and (c) taking into account that data were manually processed, future researchers may benefit from applying a software that processes information automatically, which may facilitate the understanding of the associations between records obtained from both technologies.

Overall, the present study proved the usefulness of the tri-axial accelerometer in monitoring *T. tetradactyla* activity patterns, especially in evaluating the effect of changes in environmental complexity. In addition, the importance of the validation and calibration of the accelerometer in captivity was proven, similarly to other studies, before applying this methodology in other environments, such as the wild [36,60,61]. Hopefully, further studies on zoo-housed lesser anteaters should assess the long-term effect of vests on behavioural activity and skin health, before results may serve as a basis for researchers studying the behaviour of free-ranging lesser anteaters. Finally, our multidisciplinary approach could be improved by developing semi-automatic or automatic data processing that maximizes the potential of the accelerometer.

## 5. Conclusions

In the present study, by evaluating the behaviour of adult *Tamandua tetradactyla* individuals using a tri-axial accelerometer, it was found that:

- The use of a vest for mounting the accelerometer did not influence the total activity or activity pattern.
- The pattern of fluctuations in the accelerometer validated by video recordings made it possible to clearly discriminate resting behaviour from active behaviours.
- The use of the accelerometer was biologically relevant to the characterization of the response to changes in environmental complexity, employing ODBA variance as a parameter.

In this study, the use of the accelerometer placed in a vest was demonstrated to be a reliable method to determine the activity patterns of lesser anteaters under semi-controlled conditions. Considering that zoo-housed lesser anteaters exhibit diurnal and nocturnal activity patterns, this study contributes to finding a strategy to easily monitor their activities and responses to different management procedures supporting welfare programs, as well as ex situ conservation.

## Data Availability

The data presented in this study are available upon reasonable request from the corresponding authors.

## Supplementary Materials

The following supporting information can be downloaded at: https://www.mdpi.com/article/10.3390/ani12192516/s1, Table S1: Descriptive statistics obtained for the X, Y, Z axes and the ODBA according to the behaviours observed in *Tamandua tetradactyla* individuals during 20-minute test at the experimental room and 24 h complementary test at the enclosure.
