# Supplementary material for: Video Validation of Tri-Axial Accelerometer for Monitoring Zoo-Housed Tamandua tetradactyla Activity Patterns in Response to Changes in Husbandry Conditions
Source: Animals (Basel). 2022 Sep 21;12(19):2516. doi: 10.3390/ani12192516 (PMC9559380; doi:10.3390/ani12192516)
Supplement: Supplementary file 1 [file animals-12-02516-s001.zip › animals-1876999-Supplementary Table S1.pdf]

Table S1. Descriptive statistics obtained for the X, Y, Z axes and the ODBA according to the behaviours observed in *Tamandua tetradactyla* individuals during 20-minute test at the experimental room and 24h complementary test at the enclosure..

BEHAVIOUR	Variable	n = events	n = seconds	Mean	S.D.	Var(n−1)	S.E.	C.V.	Min.	Max.	Median	Kurtosis	P(05)	P(95)
**Alert**	X	1	50	0.5413	0.3559	0.1266	0.0503	65.7456	0.0313	0.9063	0.7578	−1.7488	0.0781	0.9063
**Alert**	Y	1	50	−0.1435	0.0802	0.0064	0.0113	55.9183	−0.3281	0.1563	−0.1563	2.9936	−0.2813	−0.0313
**Alert**	Z	1	50	0.6416	0.2738	0.0750	0.0387	42.6755	0.2813	1.0625	0.4922	−1.6771	0.2813	1.0156
**Alert**	ODBA	1	50	0.2331	0.2561	0.0656	0.0362	109.8642	0.0692	1.4417	0.1474	9.9886	0.0766	0.9234
**Self-grooming**	X	167	9739	0.2910	0.2642	0.0698	0.0027	90.8199	−1.0000	1.3594	0.2656	0.8906	−0.0781	0.8125
**Self-grooming**	Y	167	9739	0.0016	0.4316	0.1863	0.0044	27525.2690	−2.1250	3.2344	0.0313	−0.1954	−0.7344	0.7031
**Self-grooming**	Z	167	9739	0.7372	0.2759	0.0761	0.0028	37.4203	−7.2969	5.0469	0.8125	88.3953	0.2500	1.0000
**Self-grooming**	ODBA	167	9739	0.3168	0.2754	0.0759	0.0028	86.9493	0.0068	12.4131	0.2532	422.4633	0.0667	0.7568
**Feeding**	X	29	20730	0.3519	0.4413	0.1947	0.0031	125.3963	−0.5313	1.0469	0.5000	−1.2201	−0.3594	0.9375
**Feeding**	Y	29	20730	−0.1135	0.2487	0.0618	0.0017	219.0930	−0.6875	0.6875	−0.1719	−1.0001	−0.4375	0.3281
**Feeding**	Z	29	20730	0.6585	0.3365	0.1132	0.0023	51.0950	−0.3750	1.0938	0.7656	0.6695	−0.0781	0.9531
**Feeding**	ODBA	29	20730	0.0636	0.0620	0.0038	0.0004	97.5035	0.0015	1.0625	0.0464	28.0721	0.0151	0.1708
**Exploration**	X	454	48080	0.2669	0.3773	0.1423	0.0017	141.3639	−1.0000	1.0625	0.1719	0.0228	−0.2344	0.9531
**Exploration**	Y	454	48080	−0.0691	0.3100	0.0961	0.0014	448.8277	−1.1094	1.0625	−0.0781	−0.2388	−0.5469	0.4531
**Exploration**	Z	454	48080	0.7221	0.3224	0.1040	0.0015	44.6512	−1.1094	3.2188	0.8594	1.7102	0.0156	0.9844
**Exploration**	ODBA	454	48080	0.2230	0.2124	0.0451	0.0010	95.2417	0.0011	3.7225	0.1568	9.5495	0.0302	0.6475
**Rest**	X	1	7080	0.7589	0.0217	0.0005	0.0003	2.8573	0.7188	0.8750	0.7500	1.3830	0.7344	0.7969
**Rest**	Y	1	7080	0.4785	0.0473	0.0022	0.0006	9.8846	0.2344	0.5313	0.5000	2.6766	0.3594	0.5156
**Rest**	Z	1	7080	0.3065	0.0300	0.0009	0.0004	9.7750	0.2188	0.4531	0.2969	3.1404	0.2656	0.3750
**Rest**	ODBA	1	7080	0.0266	0.0403	0.0016	0.0005	151.7466	0.0016	0.7906	0.0224	266.2165	0.0062	0.0474
**Motionless**	X	22	1810	0.2133	0.1313	0.0172	0.0031	61.5530	−0.4688	0.9844	0.2031	11.1194	0.0000	0.4375
**Motionless**	Y	22	1810	0.1191	0.2107	0.0444	0.0050	176.9160	−0.5938	0.7500	0.1406	1.9340	−0.2656	0.5781
**Motionless**	Z	22	1810	0.8794	0.1171	0.0137	0.0028	13.3212	0.1406	1.1094	0.9063	14.3970	0.5938	0.9531
**Motionless**	ODBA	22	1810	0.0585	0.1045	0.0109	0.0025	178.5145	0.0036	1.0740	0.0282	28.0756	0.0094	0.2302
**BEHAVIOUR**	**Variable**	**n = events**	**n = seconds**	**Media**	**S.D.**	**Var(n−1)**	**S.E.**	**CV**	**Min.**	**Max.**	**Median**	**Kurtosis**	**P(05)**	**P(95)**
**Locomotion**	X	273	18941	0.1189	0.1926	0.0371	0.0014	162.0215	−0.8281	2.3281	0.0781	6.0481	−0.0938	0.5000
**Locomotion**	Y	273	18941	−0.0221	0.3322	0.1103	0.0024	1504.7685	−1.2656	1.1250	−0.0156	−0.6732	−0.5625	0.5156
**Locomotion**	Z	273	18941	0.8603	0.1713	0.0293	0.0012	19.9096	−3.9063	2.6250	0.8906	40.3679	0.5781	1.0469
**Locomotion**	ODBA	273	18941	0.2998	0.2044	0.0418	0.0015	68.1871	0.0042	7.9516	0.2605	108.8756	0.0699	0.6542
**Others**	X	2	2540	0.6802	0.3015	0.0909	0.0060	44.3222	−0.5938	1.0625	0.7969	2.3689	0.0000	0.9688
**Others**	Y	2	2540	0.0737	0.2958	0.0875	0.0059	401.3611	−0.5781	1.0000	−0.0156	1.3418	−0.2656	0.8281
**Others**	Z	2	2540	0.1612	0.5298	0.2807	0.0105	328.6722	−0.8125	1.0781	0.2188	−1.6509	−0.5156	0.8438
**Others**	ODBA	2	2540	0.1387	0.1384	0.0191	0.0027	99.7870	0.0041	1.2244	0.1026	13.4275	0.0188	0.3994
**Climbing up**	X	36	4550	0.7794	0.2451	0.0601	0.0036	31.4446	−0.2813	1.0938	0.8750	1.9272	0.2031	0.9844
**Climbing up**	Y	36	4550	−0.1161	0.2596	0.0674	0.0038	223.5425	−1.0000	0.6563	−0.1094	0.3559	−0.5625	0.2969
**Climbing up**	Z	36	4550	0.1829	0.4266	0.1820	0.0063	233.2381	−0.8281	1.0625	0.1094	−0.9038	−0.4688	0.9063
**Climbing up**	ODBA	36	4550	0.2552	0.2269	0.0515	0.0034	88.9355	0.0053	1.7625	0.1779	4.2596	0.0422	0.7260
**Climbing down**	X	39	5500	−0.5631	0.3763	0.1416	0.0051	66.8304	−2.0000	0.6406	−0.6875	−0.2827	−0.9688	0.1563

**Climbing down**	Y	39	5500	0.0130	0.3923	0.1539	0.0053	3025.7345	−1.0469	1.0000	0.0156	0.1506	−0.7813	0.6250
**Climbing down**	Z	39	5500	0.5090	0.3021	0.0912	0.0041	59.3417	−1.0000	1.5469	0.4844	−0.8161	0.0469	0.9531
**Climbing down**	ODBA	39	5500	0.3217	0.2758	0.0761	0.0037	85.7435	0.0083	3.9479	0.2444	9.3853	0.0489	0.8796
**Repetitive locomotion***	X	14	1320	0.0399	0.1094	0.0120	0.0030	274.4660	−0.2813	0.4063	0.0313	0.0702	−0.1406	0.2344
**Repetitive locomotion***	Y	14	1320	−0.0239	0.4233	0.1792	0.0117	1771.4984	−0.8594	0.6250	0.1641	−1.5752	−0.6563	0.4844
**Repetitive locomotion***	Z	14	1320	0.8580	0.0857	0.0073	0.0024	9.9909	0.2344	1.1406	0.8594	3.2793	0.7188	0.9844
**Repetitive locomotion***	ODBA	14	1320	0.2035	0.1058	0.0112	0.0029	51.9774	0.0078	0.8948	0.1875	2.1346	0.0615	0.3990
**Inverted climbing***	X	17	2820	−0.0221	0.1685	0.0284	0.0032	760.7471	−0.5000	1.0156	−0.0313	1.8286	−0.2813	0.2656
**Inverted climbing***	Y	17	2820	0.1609	0.3431	0.1177	0.0065	213.1878	−0.9531	1.0000	0.1563	−0.3746	−0.3906	0.6875
**Inverted climbing***	Z	17	2820	−0.9251	0.1423	0.0203	0.0027	15.3836	−1.2813	0.0781	−0.9688	9.0103	−1.0625	−0.6719
**Inverted climbing***	ODBA	17	2820	0.2734	0.1695	0.0287	0.0032	61.9956	0.0078	1.2359	0.2375	4.3488	0.0770	0.5844

*Behaviours observed and characterized by the data obtained in the 24-hour test.

S.D.: Standard deviation; Var(n−1): Sample variance; S.E: standard error; CV: coefficient of variation; Min.: Minimum; Max.: Maximum; P(05): Percentile 05; P(95): Percentile 95.
